# The Association of Hypertension with Obesity and Metabolic Abnormalities among Chinese Children

**DOI:** 10.4061/2011/987159

**Published:** 2011-12-01

**Authors:** Haiquan Xu, Xiaoqi Hu, Qian Zhang, Songming Du, Hongyun Fang, Ying Li, Jun Ma, Tingyu Li, Lin Du, Hongwei Guo, Guifa Xu, Ailing Liu

**Affiliations:** ^1^National Institute for Nutrition and Food Safety, Chinese Center for Disease Control and Prevention, Beijing 100050, China; ^2^Haerbin Medical University, Heilongjiang, Haerbin 150081, China; ^3^Institute of Children and Adolescent Health, Peking University Health Science Center, Beijing 100191, China; ^4^Chongqing Children's Hospital, Chongqing 400014, China; ^5^Guangzhou Center for Disease Control and Prevention, Guangdong, Guangzhou 510515, China; ^6^School of Public Health, Fudan University, Shanghai 200032, China; ^7^Department of Public Health, Shandong University, Shandong, Jinan 250012, China

## Abstract

A total of 8898 Chinese children (4580 boys and 4318 girls) aged 7–13 years in 6 cities of east China were recruited. Data on height, weight, waist circumference, blood pressure, serum lipid profiles, glucose, and insulin were collected. The overall prevalence of hypertension was 11.1%. Overweight and obese children had a higher risk of developing hypertension than their counterparts (29.1%, 17.4%, and 7.8%, resp.) (*P* = 0.0001). The means levels of triglycerides, glucose, insulin, and HOMA-IR (1.0 mmol/L, 4.5 mmol/L, 8.4 mU/mL and 1.7, resp.) among hypertensive children were all significantly higher than their normotensive counterparts (0.8 mmol/L, 4.5 mmol/L, 5.9 mU/mL, and 1.2, resp.) (*P* = 0.0001). Compared with the healthy children, the risk (odds ratio, OR) of having hypertension among children with high triglycerides, hyperglycemia, and metabolic syndrome was 1.4 (95% confidence interval (CI): 1.0–2.0, *P* = 0.0334), 1.5 (95% CI: 0.9–2.5, *P* = 0.0890), and 2.8 (95%CI: 1.5–5.4, *P* = 0.0014), respectively, after controlling for age, gender, BMI, income level, parents' education level and puberty. In conclusion, overweight and obese children have higher risk of having hypertension and children with dyslipidemia, hyperglycemia, and metabolic syndrome and higher HOMA-IR have higher risk of developing hypertension.

## 1. Introduction

Hypertension is a major public health problem worldwide. In China, the prevalence of hypertension was 17.7% in 2002 and it was estimated that the number of adults with hypertension was 174 million and 220 thousands died from hypertension annually, respectively. About 2.54 million life years might be lost potentially due to hypertension. Every hypertensive patient led about 11.4 years life lost on average. Moreover, the direct economic burden reached 31.89 billion RMB [1]. It is well known that hypertension is one of the major risk factors for coronary artery disease and cerebrovascular disease [2, 3].

Development of adult hypertension and the adverse effects of elevated blood pressure on vascular structure and function may start very early in life [4–8]. Obesity is well known as one predictor of hypertension [9]. Some studies also showed the relationship between hypertension and cholesterol, glucose, insulin, and insulin resistance [10, 11]. However, these relationships appear to vary among ethnic groups [12]. The data on the relationship between hypertension and lipid profiles, glucose, insulin, and insulin resistance among Chinese children were limited. Therefore, the purpose of the present study was to explore the association of hypertension with obesity, metabolic abnormities including dyslipidemia, hyperglycemia, hyperinsulinemia, and metabolic syndrome among Chinese children. 

## 2. Materials and Methods

### 2.1. Participants

Multi-stage randomly cluster sampling method was used to recruit participants. Firstly, six provincial capital cities in Eastern China, including Haerbin, Beijing, Jinan, Shanghai, Chongqing, and Guangzhou were selected for this study. From each city, 6 schools were then selected randomly. Lastly, 2 classes from each grade were selected randomly in 1st–5th grade of each school, and all students in the two classes were involved in the study. A total of 9866 children aged 7–13 years were recruited and 8898 participants (4580 boys and 4318 girls) gave both blood samples and physical measurements. The information on education and income level of the parents was collected from parents by using a questionnaire. The information on puberty was surveyed by the face to face inquiry to the participants.

The protocol of the survey was approved by the Ethical Committee of the National Institute for Nutrition and Food Safety, Chinese Center for Disease Control and Prevention. Signed consent forms were obtained from both the children's parent(s) or guardian and the children themselves.

### 2.2. Anthropometric Measurements

Height was measured in bare feet to 0.1 cm with a freestanding stadiometer mounted on a rigid tripod (GMCS-I, Xindong Huateng Sports Equipment Co. Ltd., Beijing, China). Fasting body weight was measured to the nearest 0.1 kg on a double ruler scale (RGT-140, Wujin Hengqi Co. Ltd., Changzhou, China) with participants wearing lightweight clothing. BMI was calculated as weight in kilograms divided by height in meters squared (kg/m^2^). Using age- and sex-specific BMI cutoff points developed by the working group for obesity in China (WGOC), normal weight was defined as BMI for age and sex ≤85th percentile and overweight was defined as BMI between the 85th and the 95th percentiles, whereas obesity was defined as BMI ≥ 95th percentile [13]. Waist circumference (WC) was measured to the nearest 0.1 cm at the midway between the lower rib margin and the iliac crest with flexible anthropometric tape (Myotape). The WC was measured twice, and if the variation between these two measurements was greater than 2 cm, a third measurement was taken and the mean was calculated by using the two closest measurements. Height, weight, and WC were all objectively measured by trained investigators following standard procedures.

### 2.3. Blood Pressure and the Definition of Hypertension

Blood pressure was measured to the nearest 2 mmHg in the seated position using a mercury sphygmomanometer by trained nurses with at least a 10 min rest period before the measurement. The first and fifth Korotkoff sounds were used to represent the systolic (SBP) and diastolic blood pressure (DBP), respectively. Three measurements were taken from all the participants at 2 min intervals, and the average of the last two measurements was recorded. Hypertension was defined as SBP and/or DBP ≥95th age- and gender-specific percentile developed by Mi et al. for Chinese children and adolescents [14].

### 2.4. Serum Glucose, Insulin, and Lipid Profiles

Fasting blood sample (5 mL) of teach participant was collected in the morning after approximately 10–14 hours of overnight fasting. Serum glucose was determined by a glucose-oxidase method (Daiichi Pharmaceutical Co., Ltd., Tokyo, Japan) within 4 hours after the fasting blood sample was obtained. Total cholesterol (TC), triglycerides (TG), low-density lipoprotein cholesterol (LDL-C), and high-density lipoprotein cholesterol (HDL-C) were determined by enzymatic methods using commercial kits (Daiichi Pharmaceutical Co., Ltd., Tokyo, Japan). Serum insulin was determined by the AxSYM based on the microparticle enzyme immunoassay technology. The homeostatic index for insulin resistance (HOMA-IR) was calculated according to the homeostatic model as HOMA-IR = insulin fasting (*μ*U/mL) × glycemia fasting (mmol/L)/22.5 [15, 16].

### 2.5. Definition of Metabolic Abnormalities

According to the International Diabetes Federation (IDF) definition [17]. Metabolic syndrome was diagnosed by abdominal obesity (≥90th percentile as assessed by WC with cutoff values from Chinese children [18]) and the presence of two or more clinical features including high triglycerides (TG ≥ 1.7 mmol/L), low HDL-C (HDL-C < 1.03 mmol/L), elevated blood pressure (SBP ≥ 130 mmHg or DBP ≥ 85 mmHg), and hyperglycemia (fasting glucose ≥ 5.6 mmol/L).

### 2.6. Statistical Analysis

The prevalence of hypertension was estimated overall by sex, age, and BMI. Prevalence values were compared using generalized linear mixed model (GLMMIX). Considering the complex study design, generalized linear mixed model was used to compare the fixed effect on the likelihood of hypertension after controlling for random effects. The fixed effects included sex, age, BMI status, income level, parents' education level, and puberty, and the class was taken as random effect. The means of continuous variables were compared using mixed linear model (MIXED). All statistical analyses were done with SAS (9.2e for Windows; SAS Institute Inc. Cary, NC, USA), and the significance level was set at 0.05.

## 3. Results

The characteristics of the participants are shown in Table 1. Height, weight, BMI, and WC were higher among children with hypertension than children with normal blood pressure. Hypertensive children had a higher level of TG, glucose, insulin, and HOMA-IR than their normotensive counterparts after adjustment for age, gender, BMI, income level, parents' education level, and puberty (*P* < 0.0001).

The overall prevalence of hypertension among Chinese children was 11.1%. Compared with the normal-weight children, overweight and obese children had a higher risk for developing hypertension. The odds ratio (OR) was 2.9 (95% CI: 2.3–3.6) and 6.0 (95% CI: 4.9–7.4) for overweight and obese children, respectively. The prevalence of hypertension among abdominal obese children was higher than their counterparts (27.9% versus 8.4%, OR = 4.6, 95% CI: 3.8–5.5) (Table 2).

Compared with their counterparts, children with high triglycerides (OR = 1.4, 95% CI: 1.0–2.0, *P* = 0.0334), hyperglycemia (OR = 1.5, 95% CI: 0.9–2.5, *P* = 0.0890), low HDL-C (OR = 0.9, 95% CI: 0.6–1.2, *P* = 0.4507), and metabolic syndrome (OR = 2.8, 95% CI: 1.5–5.4, *P* = 0.0014) had a higher risk for having hypertension after controlling for the age, gender, and BMI. The prevalence of hypertension was higher at higher insulin and HOMA-IR values (*P* < 0.0001 in each); the odds ratio was 1.6 (95% CI: 1.4–2.0) and 1.6 (95% CI: 1.4–2.0) after controlling for age, sex, BMI, income level, parents' education level, and puberty, respectively (Table 3).

## 4. Discussion 

Our study showed that the prevalence of hypertension increased with the weight status among Chinese children and the risk of having hypertension for obese children or abdominal obese children was higher than normal-weight children. These findings were consistent with previous studies in South Asian children [19, 20] and Caucasian children [21–23]. Obesity in childhood is one of the main predictors of hypertension in both childhood and adulthood [23]. Given the alarming increase in childhood obesity in China [24], the number of children with hypertension will increase rapidly. 

The current study also indicated that, after controlling for age, gender, and BMI, the levels of TG and glucose among hypertensive children were higher than normotensive children. Hypertensive children were more likely to have higher TG, hyperglycemia, and MetS compared with their normotensive counterparts. These findings are consistent with the previous studies in Chinese adults [25, 26] and other ethnic populations [27–29]. TG increase may promote hypertension by the development of atherosclerotic changes in blood vessels [30]. Moreover, the pre-3-lipoprotein fraction rich in endogenous triglycerides inhibits the plasminogen activator secretion in the blood vessel wall and its deficiency may cause inhibition of fibrinolysis. The fibrinolysis inhibition also can promote hypertension by the development of atherosclerotic changes in blood vessels [30]. Hyperglycemia can alter intracellular signaling pathway leading to endothelial dysfunction, which was associated with increased blood pressure [31, 32]. 

Insulin and HOMA-OR in children with hypertension was found higher than children with normal blood pressure in our study. Some previous studies indicated the positive relationship between hyperinsulinemia, insulin resistance and hypertension and/or blood pressure [33, 34]. Insulin resistance might cause compensatory hyperinsulinemia, while hyperinsulinaemia could stimulate the activity of the sympathetic nervous system and kidney sodium and volume reabsorption, which could contribute to hypertension [35]. In addition, insulin resistance has been shown to be associated with impaired endothelium-dependent vasodilatation [36], which could contribute to increase blood pressure. 

In conclusion, overweight and obese children were at a higher risk of having hypertension. Hypertensive children had a higher level of TG, glucose, insulin, and HOMA-IR. There is a higher risk of developing hypertension among children with dyslipidemia, hyperglycemia, and MetS than their counterparts. Effective interventions should be targeted for Chinese children at the onset of childhood obesity and hypertension. 

##  Authors contribution

Y. Li, J. Ma, T. Li, L. Du, H. Guo, and G. Xu equally contributed to the paper.

##  Conflict of Interests

None of the authors had a personal or financial conflict of interests.

## Figures and Tables

**Table 1:** The characteristics of participants (mean ± SD).

	Normotensive	Hypertensive	Total	*P*
Age (years)	8.7 ± 1.5	8.7 ± 1.4	8.7 ± 1.4	0.0306
Height (cm)	134.8 ± 9.6	138.5 ± 9.7	135.2 ± 9.7	<0.0001*
Weight (kg)	31.0 ± 8.6	36.8 ± 11.0	31.6 ± 9.1	<0.0001*
WC (cm)	57.0 ± 8.3	63.2 ± 11.0	57.7 ± 8.9	<0.0001*
BMI (kg/m^2^)	16.8 ± 2.9	18.9 ± 3.9	17.0 ± 3.1	<0.0001*
TG (mmol/L)	0.8 ± 0.4	1.0 ± 0.5	0.8 ± 0.4	<0.0022**
TC (mmol/L)	4.1 ± 0.8	4.2 ± 0.8	4.1 ± 0.8	0.1127**
HDL-C (mmol/L)	1.5 ± 0.3	1.4 ± 0.3	1.5 ± 0.3	0.3937**
LDL-C (mmol/L)	2.1 ± 0.6	2.2 ± 0.6	2.1 ± 0.6	0.3260**
Glucose (mmol/L)	4.5 ± 0.6	4.5 ± 0.6	4.5 ± 0.6	<0.0001**
Insulin (mU/mL)	5.9 ± 4.2	8.4 ± 6.7	6.1 ± 4.6	<0.0001**
HOMA-IR	1.2 ± 0.9	1.7 ± 1.6	1.3 ± 1.0	<0.0001**

* Adjusted for age, gender, income level, parents' education level, and puberty.

** Adjusted for age, gender, BMI, income level, parents' education level, and puberty.

**Table 2:** The prevalence of hypertension in Chinese children by weight status.

	*N* (%)	OR (95% CI)	*P*
Total (*n* = 8898)	985 (11.1)	—	—
Weight status			
Normal weight (*n* = 6953)	539 (7.8)	1	—
Overweight (*n* = 1019)	177 (17.4)	2.9 (2.3–3.6)	<0.0001
Obesity (*n* = 926)	269 (29.1)	6.0 (4.9–7.4)	<0.0001
Abdominal obesity			
No (*n* = 7696)	650 (8.4)	1	—
Yes (*n* = 1202)	335 (27.9)	4.6 (3.8–5.5)	<0.0001

Adjusted for age, gender, income level, parents' education level, and puberty.

**Table 3:** The prevalence of hypertension among children with metabolic abnormalities and normal children.

	No (%)	OR	Yes (%)	OR (95% CI)	*P*
High TG	10.6	1	23.3	1.4 (1.0–2.0)	0.0334
Low HDL-C	11.0	1	12.6	0.9 (0.6–1.2)	0.4507
Hyperglycemia	11.0	1	15.5	1.5 (0.9–2.5)	0.0890
MetS	10.8	1	51.9	2.8 (1.5–5.4)	0.0014
Insulin	7.7	1	16.6	1.6 (1.4–2.0)	<0.0001
HOMA-IR	7.5	1	15.4	1.6 (1.4–2.0)	<0.0001

Adjusted for age, gender, BMI, income level, parents' education level, and puberty.

The cutoff point was median for insulin and HOMA-IR.
